# Aggressive migration in acidic pH of a glioblastoma cancer stem cell line in vitro is independent of ASIC and K_Ca_3.1 ion channels, but involves phosphoinositide 3-kinase

**DOI:** 10.1007/s00424-022-02781-w

**Published:** 2022-12-16

**Authors:** Klaus-Daniel Cortés Franco, Ilka C. Brakmann, Maria Feoktistova, Diana Panayotova-Dimitrova, Stefan Gründer, Yuemin Tian

**Affiliations:** 1 Institute of Physiology, RWTH Aachen University, Pauwelsstraße 30, D-52074 Aachen, Germany; 2 Department of Dermatology, RWTH Aachen University, Pauwelsstraße 30, D-52074 Aachen, Germany

**Keywords:** Acid sensing ion channel, Glioblastoma multiforme, Extracellular acidity, Tumour microenvironment, Tumour spheroids

## Abstract

The microenvironment of proliferative and aggressive tumours, such as the brain tumour glioblastoma multiforme (GBM), is often acidic, hypoxic, and nutrient deficient. Acid-sensing ion channels (ASICs) are proton-sensitive Na^+^ channels that have been proposed to play a role in pH sensing and in modulation of cancer cell migration. We previously reported that primary glioblastoma stem cells (GSCs), which grow as multicellular tumour spheroids, express functional ASIC1a and ASIC3, whereas ASIC2a is downregulated in GSCs. Using a 2.5D migration assay, here we report that acidic pH dramatically increased migration of GSCs of the pro-neural subtype. Pharmacological blockade as well as CRISPR-Cas9-mediated gene knock-out of ASIC1a or stable overexpression of ASIC2a, however, revealed that neither ASIC1a nor ASIC3, nor downregulation of ASIC2a, mediated the aggressive migration at acidic pH. Therefore, we tested the role of two other proteins previously implicated in cancer cell migration: the Ca^2+^-activated K^+^ channel KCa3.1 (KCNN4) and phosphoinositide 3-kinase (PI3K). While pharmacological blockade of K_Ca_3.1 did also not affect migration, blockade of PI3K decreased migration at acidic pH to control levels. In summary, our study reveals a strongly enhanced migration of GSCs at acidic pH in vitro and identifies PI3K as an important mediator of this effect.

## Introduction

Glioblastoma, IDH wild-type multiforme (GBM) is a type of glioma. GBM is the most common primary malignant brain tumour, with a survival rate of < 10% 5 years after the initial diagnosis [53]. Surgical resection, followed by radiotherapy and chemotherapy, is the most common treatment [46]; however, due to the infiltration of GBM into the surrounding brain tissue, the likelihood of tumour recurrence and eventual radio- and chemoresistance is > 86% [37]. GBM is a rapidly proliferating, aggressive, invasive, and undifferentiated type of tumour [17, 53] which uses glycolysis for ATP production, despite oxygen being available for oxidative phosphorylation [38]. This aerobic glycolysis, termed the Warburg effect, together with insufficient blood supply, acidifies the tumour microenvironment via the fermentation of pyruvate to lactate. An acidic microenvironment has been recognized to be relevant for proliferation, survival, metabolic adaptation, and migration in cancer [45, 51]. However, the mechanism of enhanced migration of cancer cells at acidic pH is insufficiently understood [7].

Acid-sensing ion channels (ASICs) are proton-gated Na^+^ channels, which, in the CNS, assemble either as homomeric ASIC1a or heteromeric ASIC1a/2a or ASIC1a/2b [52]. It has been shown that functional homomeric ASIC1a is also expressed in glioblastoma stem cell lines (GSCs) [47]. These GSCs grow as tumour spheres and are cultured without serum, because serum causes irreversible differentiation of GSCs [12]. GSCs closely recapitulate the genotype, gene expression pattern, and in vivo biology of primary tumours [29], whereas serum-cultured glioma cell lines do not [29, 30]. ASIC2a, in contrast, is downregulated in GSCs and in GBM tissue [47]. Thus, it is conceivable that ASIC1a senses acidosis in GBM. ASICs have indeed been implicated in pH sensing and migration of glioma cells [24, 40, 43, 49]. However, previous assays assessing the role of ASICs in migration in GBM cell cultures have been performed with serum-cultured glioma cell lines using transwell or wound-healing assays [24, 40, 43, 49].

Phosphoinositide 3-kinases (PI3K) are members of the PI3K/Akt/MTOR pathway, which is often mutated in cancer cells. PI3K is also known to be activated by hypoxia [55] and is linked to proliferation, cell survival, differentiation, migration, and poor disease prognosis in patients [32]. KCa3.1 (KCNN4) is an intermediate-conductance Ca^2+^-activated K^+^ channel (also known as IKCa) activated by intracellular Ca^2+^. It contributes to controlling the cell membrane potential [25] and has been reported to play a role in migration and proliferation of GBM [6, 42], hepatocellular carcinoma [31, 33], and migration in colorectal cancer [27], lung adenocarcinoma [54], and pancreatic cancer cells [18].

Here, we established a 2.5D migration assay and show much more aggressive migration of GSCs at an acidic extracellular pH (pH_e_). While we found no evidence for the involvement of ASICs or KCa3.1 in enhanced migration, blockade of PI3K abrogated enhanced migration at acidic pH_e_, identifying PI3K as a novel mediator of the more aggressive migration at acidic pH_e_.

## Results and discussion

### Characterization of pH-dependent migration in multicellular tumour GSCs

**Fig. 1:**
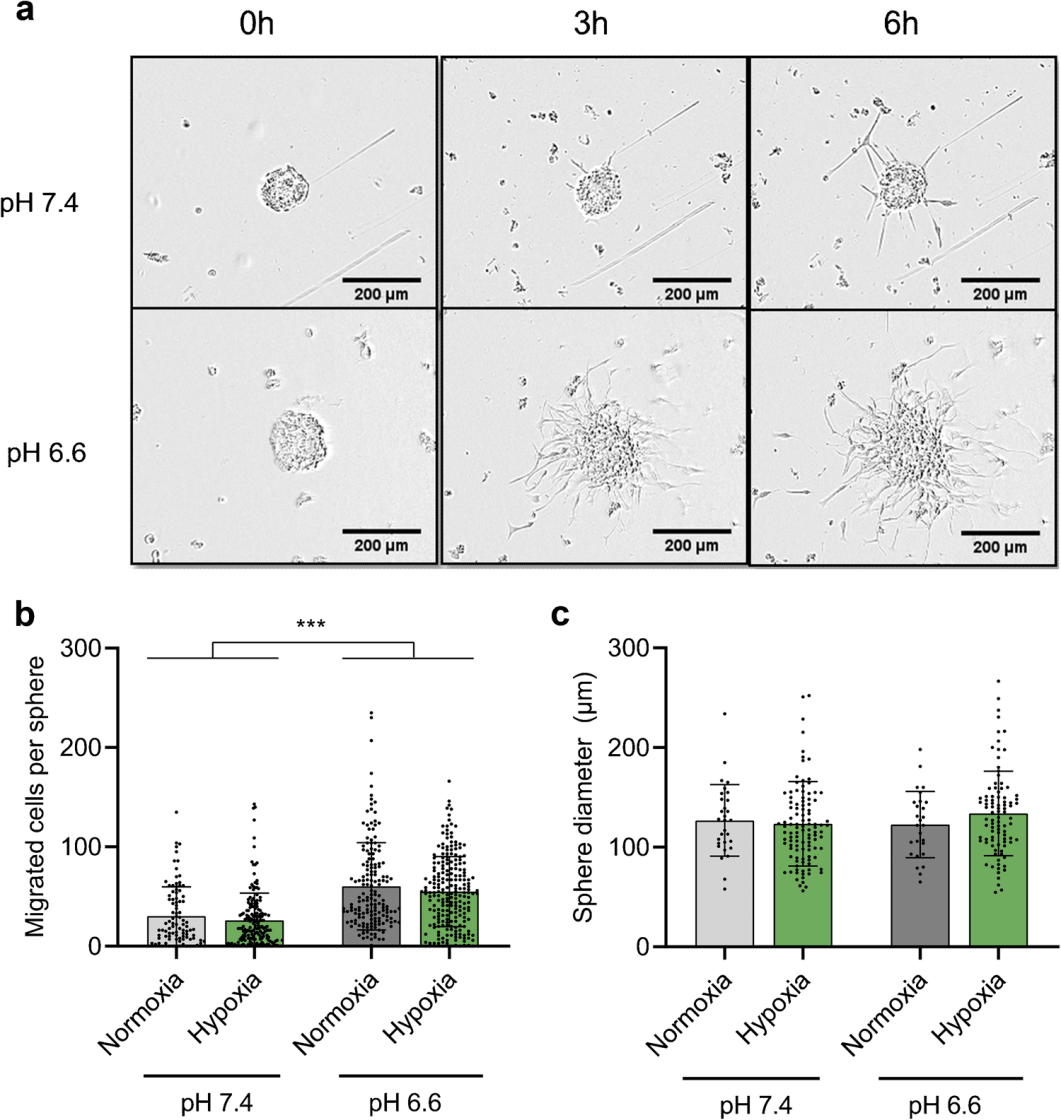
Sphere migration strongly increases at pH 6.6, independent of oxygenation. **a** Representative pictures of 7-day-old spheres incubated at pH 7.4 or pH 6.6 on laminin-coated plates, with pictures taken every three hours. Scale bars = 200 µm. **b** Comparison of sphere migration under hypoxia (O2 < 3%) and normoxia. *n* (from left to right) = 176, 96, 221, 165 individual spheres from 12 biological replicates for hypoxia and three biological replicates for normoxia. ***p < 0.001 (Tukey’s test). **c** Sphere diameters. n (from left to right) = 109, 30, 93, 27. For sphere diameters, we did not account for variations in the number of technical replicates. Bars represent mean ± SD

We established a sphere migration assay, which allowed us to test the migration of R54 cells, CD133^+^, pro-neural-like GSCs [35], in medium with a pH of 7.4 or 6.6. An acidic pH of 6.6 was chosen, as it is a relatively low pH value that can still be reached in GBM tissue [13]; in addition, it leads to robust activation of ASIC1a [2, 50]. Migration of GSCs out of spheres strongly increased at acidic pH_e_ (Fig. 1a; *p* < 0.001). Culturing the spheres under normoxic or hypoxic (< 3% O2) conditions did not affect migration (Fig. 1b), which was unexpected, as numerous publications reported increased migration under hypoxic conditions [9, 20, 23]. It is possible that this was because the cores of the spheres were already hypoxic. Moreover, sphere sizes were the same between conditions (Fig. 1c). Thus, the profound increase in migration at pH 6.6 was driven by extracellular acidity and not by hypoxia, allowing for the specific assessment of acidity on migration.

### ASIC1, ASIC2, and ASIC3 do not modulate pH-dependent migration

**Fig. 2:**
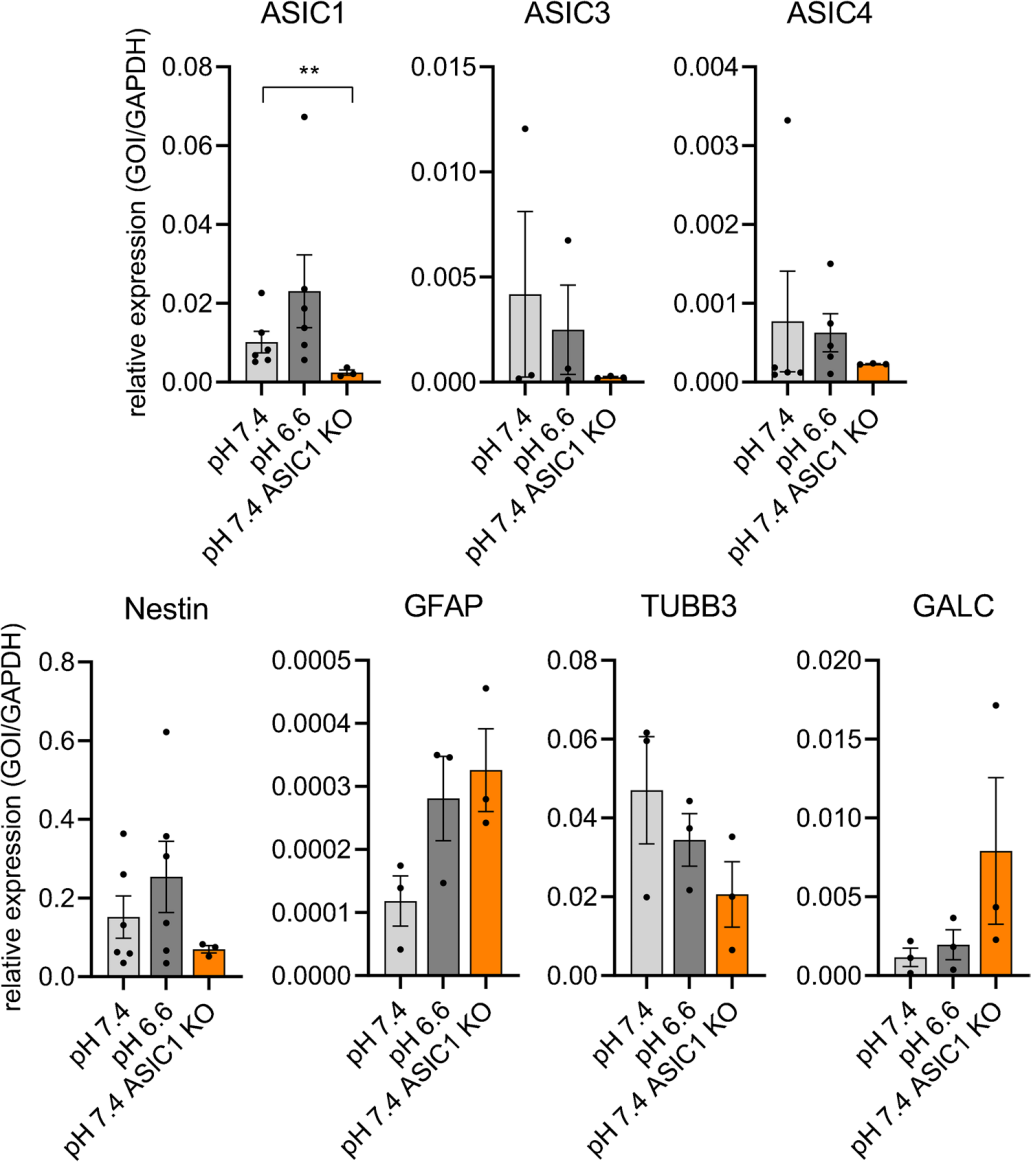
RNA expression of ASICs, stemness, and differentiation markers in R54 cells. Expression of ASIC1, ASIC3, ASIC4, stemness marker nestin, and the differentiation markers GFAP, TUBB3, and GALC, normalized to the expression of GAPDH. Dots represent biological replicates, which were at least *n* = 3 for every condition, with three technical replicates for each biological replicate. Bars represent mean ± SEM. GOI, gene of interest. **p < 0.01 (two-sided *t* test of ΔCt-values)

We assessed the expression of *ASIC1*, *ASIC2*, *ASIC3*, and *ASIC4* genes using quantitative real-time PCR (qPCR). We found that ASIC1 was most strongly expressed and that ASIC3 and ASIC4 were expressed at two- to tenfold lower levels (Fig. 2), whereas ASIC2 was not expressed. These findings are in accordance with those of a previous study [47] and microarray data from a large cohort of glioma samples [14]. We did not find significant differences in expression in R54 cells maintained at pH 6.6 for 3 days (Fig. 2). We also tested mRNA expression in a recently described R54 cell line with an ASIC1a knockout [5] (Fig. 2). Compared to WT R54 cells, ASIC1 signal was still present, but was significantly lower (*p* = 0.0086) in ASIC1a knockout cells, likely due to the knock-out inducing a frameshift in the second exon of ASIC1a, thereby impairing the binding of the qRT-PCR probe at the exon junction of exon 2 to exon 3.

We also assessed the expression of the stemness marker nestin and the differentiation markers glial fibrillary acidic protein (GFAP), tubulin beta 3 (TUBB3), and galactocerebrosidase (GALC), which are markers of astrocytes, neurons, and oligodendrocytes, respectively. While nestin expression was high, the expression of TUBB3 and, in particular, of GFAP and GALC was much lower. There was a tendency for increased expression of nestin and of GFAP at pH 6.6, but this change was not statistically significant (*p* = 0.65 and *p* = 0.14, respectively) (Fig. 2).

**Fig. 3:**
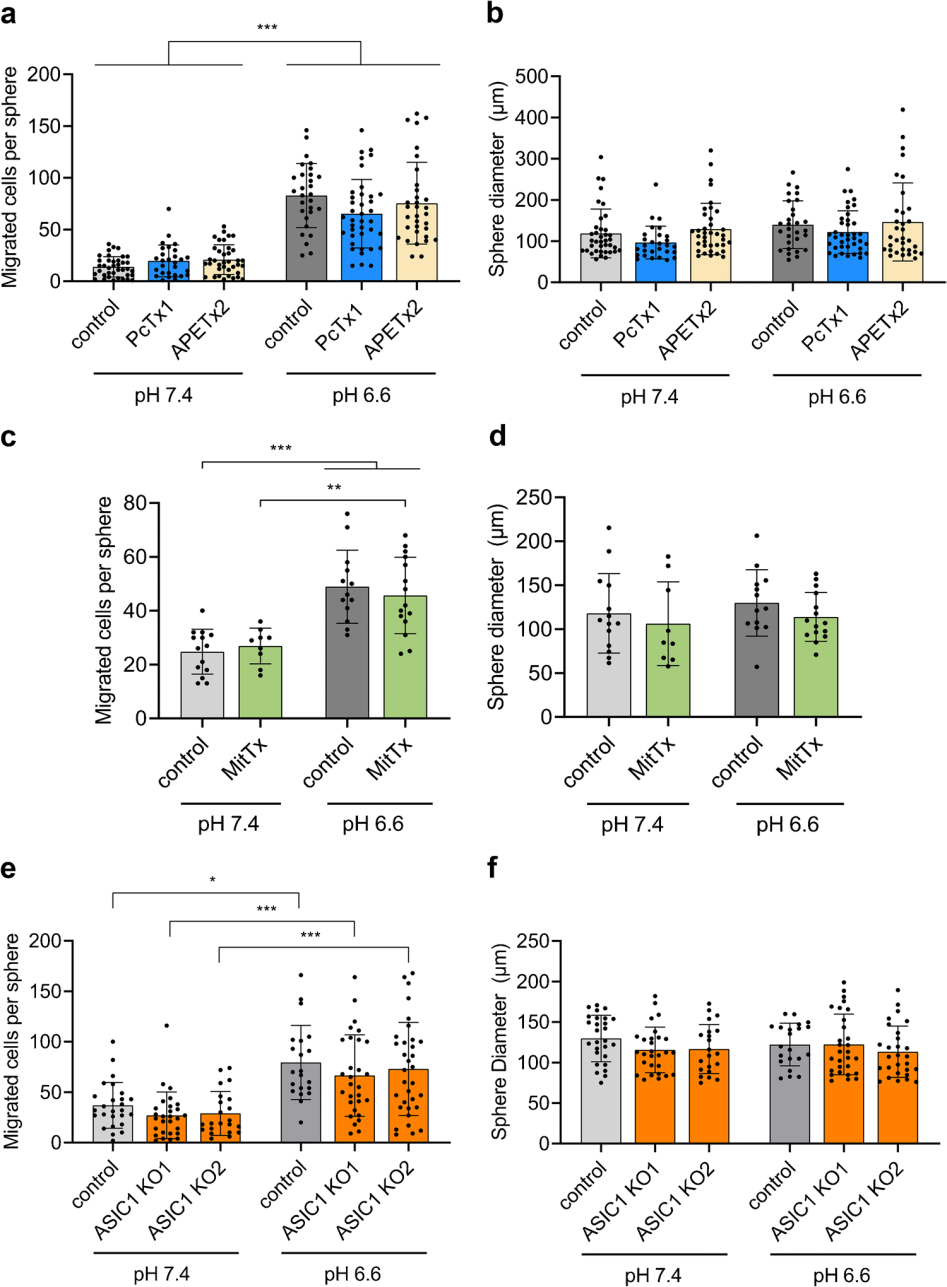
Enhanced migration of GSCs at acidic pH is not mediated by ASIC1a or ASIC3. **a** Inhibition of ASIC1 with 100 nM PcTx1 or of ASIC3 with 500 nM APETx2 did not affect migration at pH 7.4 or pH 6.6. *n* (from left to right) = 38, 28, 37, 29, 39, 33 individual spheres. **b** Sphere diameters between conditions were not significantly different from each other. **c** Activation of ASIC1a with 20 nM MitTx did not increase migration at pH 7.4 or pH 6.6. *n* (from left to right) = 14, 9, 13, 15. **d** Sphere diameters between conditions were not significantly different from each other. **e** Monoclonal R54 ASIC1a/b knockout cell lines migrated more aggressively at pH 6.6 than at pH 7.4. *n* (from left to right) = 26, 28, 22, 21, 29, 33. **f** Sphere diameters were not significantly different from each other. All experiments were performed in three biological replicates. Dots represent technical replicates. Bars represent mean ± SD. *p < 0.05, **p < 0.01, ***p < 0.001 (Tukey’s test)

To test for the involvement of ASIC1a and ASIC3 in enhanced migration at acidic pH_e_, we assessed migration of GSCs at acidic pH 6.6 in the presence of the potent ASIC1a inhibitor psalmotoxin 1 (PcTx1; 100 nM) or the potent ASIC3 inhibitor APETx2 (500 nM). Neither inhibitor had an effect on migration, irrespective of pH (Fig. 3a). They also did not affect sphere diameters (Fig. 3b). We then used the potent ASIC1 agonist MitTx (20 nM) to test whether the pharmacological activation of ASIC1 could enhance migration at neutral pH_e_. Like PcTx1, however, MitTx had no effect on migration (Fig. 3c) or on sphere diameters (Fig. 3d). Finally, we assessed the outgrowth of spheres using two independent R54 lines with an ASIC1a knockout [5]. Also ASIC1a knockout did not affect migration (Fig. 3e) or sphere diameters (Fig. 3f). These results consistently show that ASIC1a does not affect migration of R54 GSCs, neither at pH 7.4 nor at pH 6.6.

**Fig. 4:**
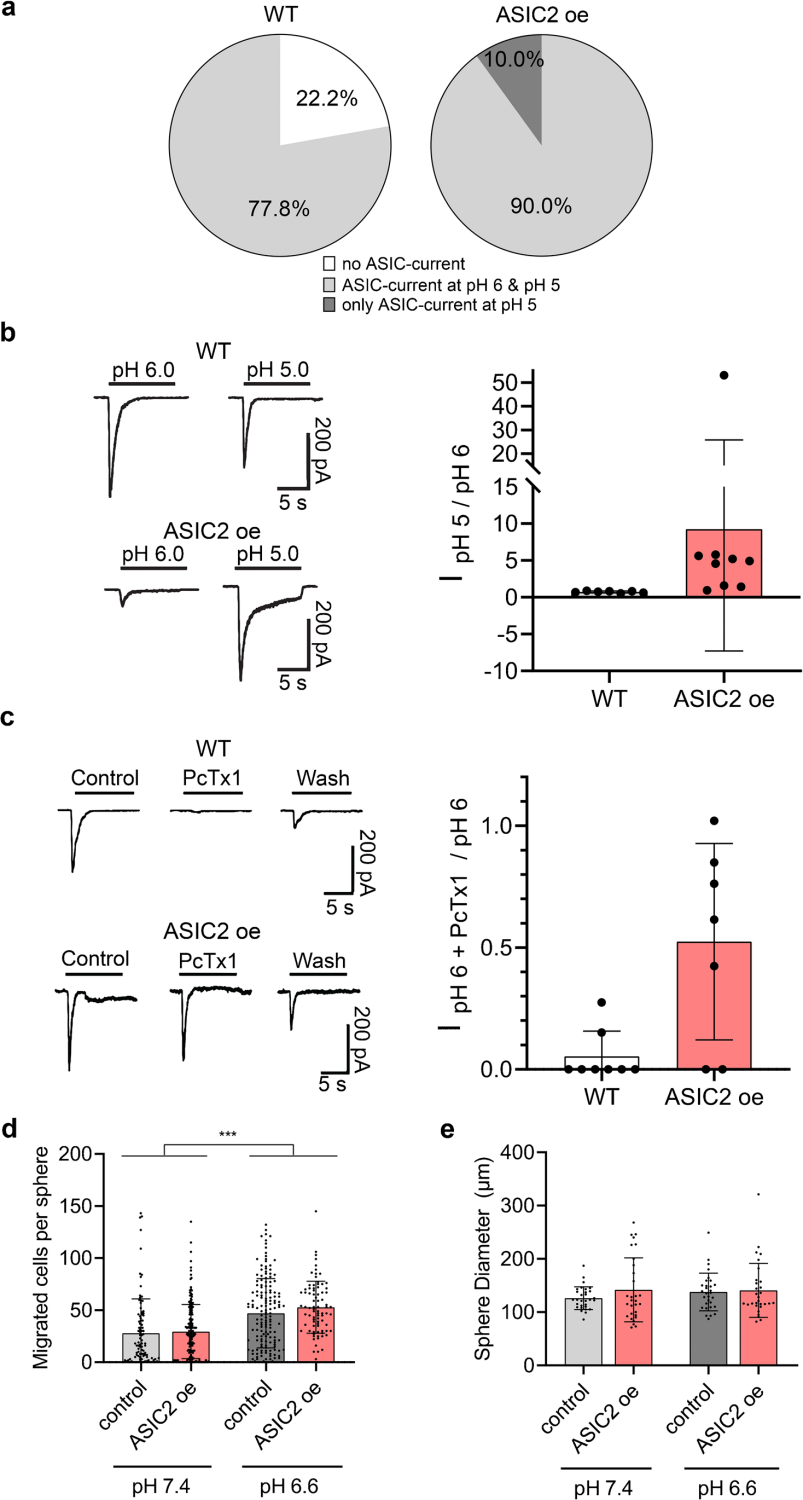
Overexpression of ASIC2a does not change aggressive migration of GSCs at acidic pH. **a** Pie chart indicating the relative occurrence of R54 cells with no ASIC current, ASIC currents elicited by pH 6 and by pH 5, and ASIC currents elicited only by pH 5. *n* = 9 for WT and *n* = 10 for ASIC2-over-expressing (oe) cells. **b** Left, representative current traces of wild-type and ASIC2 oe cells at pH 6 and pH 5. Right, ratio of ASIC currents elicited by pH 5 and pH 6. Error bars represent the mean ± SD; the mean value for ASIC2 oe cells was 9.23. *n* = 7–9. **c** Left, representative current traces of wild-type and ASIC2 oe cells at pH 6, in the absence and presence of PcTx1. Right, ratio of ASIC currents elicited by pH 6, in the presence of PcTx1. Error bars represent the mean ± SD; *n* = 7–8. **d** Sphere migration of R54 wild-type and ASIC2 oe cells. *n* (from left to right) = 99, 184, 157, 83. The experiment was performed in three biological replicates. Dots represent technical replicates. Bars represent mean ± SD. ***p < 0.001 (Tukey’s test). **e** Sphere diameters between conditions were not significantly different from each other. *n* = 30 for each condition

The downregulation of ASIC2a in GBM is striking [47], and it has been proposed that ASIC2a modulates migration of tumour cells [49, 57]. Therefore, we generated stably expressing ASIC2a R54 cells via lentiviral transduction. Electrophysiological characterization of the ASIC2a-overexpressing cells confirmed that ASIC currents in almost all cells had decreased proton sensitivity and an insensitivity to PcTx1 typical for heteromeric ASIC1a/ASIC2a [22] (Fig. 4a–e), confirming plasma membrane expression of ASIC2a in these cells. Strikingly, however, ASIC2a-overexpressing R54 cells displayed the same outgrowth as the wild-type parental cells and still aggressively migrated at an acidic pH 6.6 (Fig. 4f); sphere diameters were also unaffected (Fig. 4g). Thus, ASIC2a overexpression had no effect on migration of R54 GSCs, suggesting that ASIC2a downregulation by GBM cells did not affect migration.

### *PI3K, but not K*_*Ca*_*3.1, modulates pH-dependent migration*

**Fig. 5:**
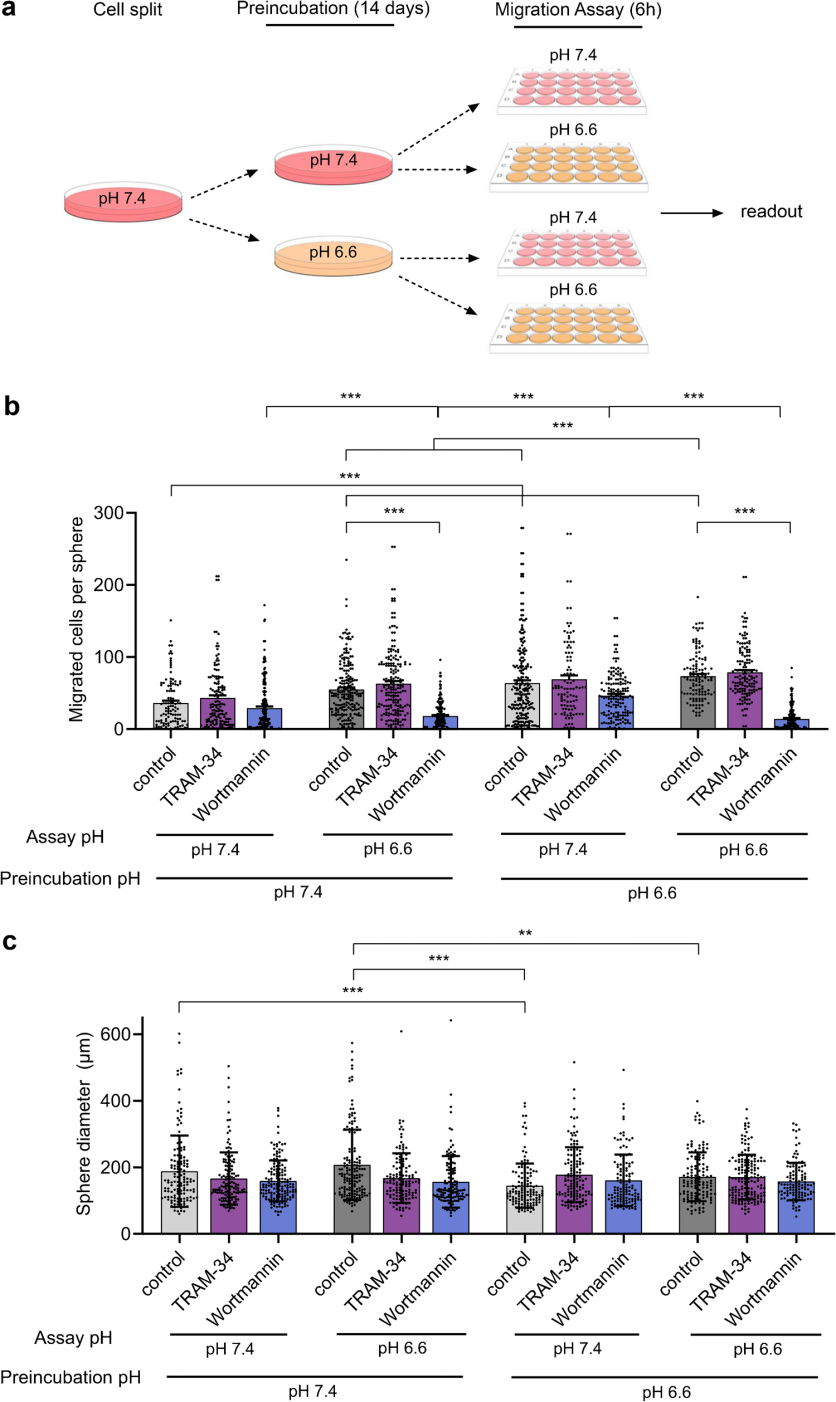
Acid-selection primes GSCs for aggressive migration and the PI3K-inhibitor wortmannin inhibits acute induction of migration by acidic pH_e_. **a** Experimental design of preincubation and assay pH conditions. Cells were grown into spheres for 14 days at either pH 7.4 or pH 6.6, and then positioned on laminin-coated wells for 6 h at pH 7.4 or pH 6.6. **b** Sphere migration with and without TRAM-34 and wortmannin. Experiments were performed in three biological replicates. Dots indicate technical replicates. *n* (from left to right) = 110, 141, 213, 208, 194, 191, 232, 101, 162, 119, 147, 198. Bars represent mean ± SD. ***p < 0.001 (Tukey’s test). **c** Sphere diameters. Dots indicate technical replicates. *n* (from left to right) = 131, 139, 159, 165, 132, 138, 136, 133, 125, 131, 162, 121. **p < 0.01, ***p < 0.001 (one-way ANOVA)

So far, we only tested the influence on migration of acutely changing pH_e_ from 7.4 to 6.6. To test whether chronic exposure to acidic pH_e_ will also affect migration of GSCs, we maintained spheres for 2 weeks either at pH 7.4 or at pH 6.6 before performing the outgrowth assay for 6 h at either pH 7.4 or pH 6.6 (Fig. 5a). The large and abrupt change in pH_e_ might favour selection of GSCs surviving at low pH rather than adaptation of GSCs to low pH [3]. Migration of cells from spheres that were maintained in acidic medium for 2 weeks was indeed strongly enhanced even when tested at pH_e_ of 7.4 (*p* < 0.001; Fig. 5b). In fact, migration of these cells at pH 7.4 was not different from that of cells maintained at pH 7.4 and tested at pH 6.6. However, cells maintained at pH 6.6 and tested at an acute pH_e_ of 6.6 showed the most aggressive migration (*p* < 0.001; Fig. 5b). Incubation of GSCs at an acidic pH might have primed them for aggressive migration or might have selected GSCs that migrated more aggressively. Sizes of spheres grown at pH 6.6 were smaller than those of spheres grown at pH 7.4 (Fig. 5b–c), which was expected, as the doubling time in pH 6.6 is longer for R54 GSCs [5]. It is unlikely, however, that the smaller sphere sizes affected the interpretation of this experiment, because, if anything, fewer cells should grow out of smaller spheres.

We then considered other targets affecting the pH-dependent migration of GSCs, and turned to K_Ca_3.1, which has been reported to play a role in glioma cell migration [4, 6, 16, 42] and GBM invasiveness in vivo [6], and to PI3K, which is a well-known modulator of migration in GBM [1, 20, 21, 34]. K_Ca_3.1 has been reported to be overexpressed in patient-derived GBM [48], whereas PI3K is overexpressed in GBM cell lines but not in patient-derived samples [56]. To assess the role of K_Ca_3.1 and PI3K, we added the K_Ca_3.1 inhibitor TRAM-34 (10 µM) or the pan-PI3K inhibitor wortmannin (1 µM) to the spheres 30 min before the start of the migration assay; we performed these assays with spheres maintained at pH 7.4 or at pH 6.6. While TRAM-34 did not affect migration under any conditions, wortmannin strongly reduced migration, but only when outgrowth was tested at pH_e_ 6.6 (Fig. 5b). In contrast, enhanced outgrowth at pH_e_ 7.4 of cells maintained at pH 6.6 was not reduced by wortmannin (*p* = 0.51). This suggests that PI3K plays an essential role in the acute induction of migration by acidic pH_e_ and that the priming of migration or selection of aggressively migrating GSCs by acidic pH is PI3K-independent and relies on a different mechanism, at least when tested at pH 7.4. The finding that wortmannin strongly reduced migration at pH_e_ 6.6 of cell maintained at acidic pH below the level of migration of these cells at pH_e_ 7.4 is not entirely consistent with this interpretation and suggests a complex interplay of different mechanisms in acid-dependent migration of GSCs.

We note that the 2.5D sphere migration assay can only partially reproduce migration of tumour cells in situ. Cancer cells in situ are likely to be confronted with slower changes in pH_e_ than the abrupt changes used in our experiments. In addition, serum-free conditions may not accurately represent the micro-environment of tumours in vivo. Nevertheless, we believe that our assay provides an important addition to earlier migration assays assessing the role of ASICs in tumour cell migration, which used serum-cultured glioma cell lines, which do not well represent the parental tumour [29, 30], in combination with wound-healing or transwell migration assays. The exact effect of acidic condition on glioblastoma cancer stem cell migration in vivo warrants further investigation.

Our findings using patient-derived R54 GSCs and a 2.5D migration assay do not confirm previous publications reporting that ASIC1, ASIC2, or ASIC3 play a role in pH- or hypoxia-dependent migration in CNS [24, 40, 43, 49], non-CNS tumours [57], and normal tissues [15]. In this respect, it is noteworthy that some of these studies assessed migration only at pH 7.4 [15, 24, 40, 49]. Mechanistically, it is unclear how a proton-gated ion channel can influence migration at neutral pH. Indeed, a recent study could not reproduce inhibition of migration by PcTx1 at pH 7.4 in U87MG GBM cells [43], as it had previously been reported for the unspecific ASIC blocker amiloride [49]. However, the same study found enhanced migration at pH 7.0, which could be inhibited by PcTx1 in two GBM cell lines [43]. Similarly, another study found an inhibition of enhanced migration at pH 6.4 by amiloride or by siRNA targeting ASIC1a or ASIC3 in two pancreatic cancer cell lines [58]. Moreover, overexpression of ASIC2a increased migration of colorectal cancer cells at pH 6.5, whereas ASIC2 knockdown decreased migration. Thus, there is conflicting evidence regarding the role of specific ASICs in cancer cell migration. This conflicting evidence could be due to differences in pH used in these studies [45] or to a different role of ASICs in cell lines originating from different tumours. Our study shows that ASICs do not have a universal role in migration of tumour cells.

Likewise, a role for K_Ca_3.1 has been reported in numerous studies [6, 16, 27, 31, 42, 48] but could not be reproduced by us in a 2.5D sphere migration assay in R54 GSCs. This might be due to the fact that R54 GSCs are of the pro-neural subtype [35], while it has been proposed that K_Ca_3.1 is a marker for the mesenchymal subtype of GSCs [26]. It will be worthwhile to assess in the future whether K_Ca_3.1 plays a role in acid-induced migration of mesenchymal subtype GSCs.

In contrast, our findings reveal a novel and prominent role for PI3K in acid-induced migration of R54 GSCs. Because PI3K is an intracellular enzyme, it must have been activated either by a non-ASIC proton-sensor on the surface of R54 cells or by a drop in intracellular pH that often accompanies extracellular acidification. A few previous studies already reported an activation of PI3K by acidic pH_e_: activation of PI3K is crucial for triggering fatty acid synthesis in liver cancer cells by an acidic TME [36], for activation of human neutrophils by extracellular acidosis [8], and for cardioprotection by acidic reperfusion [39]. The precise mechanisms of PI3K activation and its downstream targets in R54 cells need to be further explored in the future.

## Conclusion

In this study, using a 2.5D sphere migration assay and patient-derived R54 GSCs, we could not confirm a role for ASIC1, ASIC2, ASIC3, or K_Ca_3.1 in pH-dependent migration of GBM cells. However, wortmannin reduced migration induced by acute extracellular acidosis suggesting that PI3K plays a crucial role in pH-dependent migration of GBM cells. The mechanism by which acidic pH_e_ activates PI3K to induce migration remains unclear and needs to be elucidated in the future.

## Methods

### Cell culture

The cell line R54 [35] is an IDH1/2 WT proneural-like CD133^+^ primary patient-derived GBM stem cell (GSC) line that grows as a heterogeneous tumour sphere [29, 35]. R54 cells were grown in serum-free Dulbecco’s modified Eagle medium (DMEM):F12 (1:1; PAN-Biotech, Aidenbach, Germany) containing 1.2 g/l NaHCO_3_, supplemented with 2% Neuropan 27 (PAN-Biotech), 1% of 200 mM L-Glutamine (Thermo Fisher Scientific, Waltham, USA), 20 ng/ml recombinant human epidermal growth factor (EGF; R&D Systems, Minneapolis, USA), and 20 ng/ml recombinant human fibroblast growth factor (FGF; 154 a.a.; Thermo Fisher Scientific). They were regularly confirmed to be mycoplasma-negative via PCR. Medium with a pH of 6.6 was prepared with powdered medium not containing NaHCO_3_, which was added at a concentration of 0.4002 g/l, and also contained 15 mM HEPES, additionally to the supplements listed above. Cells were split with 0.05% Trypsin–EDTA solution (Thermo Fisher Scientific) into single cells once per week for regular cell maintenance (37 °C, 5% CO_2_).

### qRT-PCR

R54 GSCs were incubated in pH 7.4 or pH 6.6 for 3 days. RNA was then isolated from GSCs using the NucleoSpin RNA isolation kit (Macherey–Nagel, Düren, Germany), and concentration and quality were measured with the NanoDrop 2000c spectrophotometer (Thermo Fisher Scientific). RNA was reverse transcribed into cDNA with the High Capacity cDNA Reverse Transcription Kit (Thermo Fisher Scientific), according to the manufacturers’ instruction. Quantitative real-time PCR (qRT-PCR) was conducted using 20 ng of cDNA template per reaction, as well as the Luna mastermix (NEB) in combination with FAM-MGB labelled hydrolysis (TaqMan™) probes from Thermo Fisher Scientific, namely: ASIC1a (Hs00952802), ASIC1b (Lot 1,431,812), ASIC2 (Hs00153756), ASIC3 (Hs00245092), ASIC4 (Hs00539823), nestin (Hs00707120), GFAP (Hs00909233), TUBB3 (Hs00801390), and GALC (Hs00164660), with GAPDH as a housekeeping gene (Hs02758991). Reactions containing 5 µl Luna NEB mastermix, 1 µl cDNA, 1 µl TaqMan probe, and 3 µl sterile water were pipetted into 4-Strip 0.1 ml Tubes (STARLAB, Hamburg, Germany) and transferred to the Rotor-Gene Q thermocycler (QIAGEN, Hilden, Germany) for real-time measurements. Reactions were performed in technical triplicates for each biological replicate, with technical duplicate negative controls for each TaqMan probe. The qRT-PCR programme was conducted with an initial denaturation step (95 °C, 20 s), 40 cycles of denaturation (95 °C, 30 s), annealing (60 °C, 20 s), and elongation (72 °C, 20 s), followed by a final elongation step (72 °C, 2 min). Experiments were repeated with RNA isolated from at least *n* = 3 independent cell batches. The Rotor-Gene Q software (Version 1.7.87, QIAGEN) was then used for analysis. Statistical analysis was performed with ΔCt values using an unpaired two-sided Student’s *t* test, comparing R54 cells at pH 7.4 and pH 6.6, as well as R54 cells and R54 ASIC1 KO cells at pH 7.4. ΔCt values are normalized logarithmic expression values and as such expected to be normally distributed [11].

### Sphere migration assay

We used a 2.5D sphere migration assay (SMA) that relies on the attachment of spheres to laminin-coated plates and the subsequent migration of individual GSCs out of the spheres. The day before the experiment, 24-well plates (Thermo Fisher Scientific) were coated with 180 µl laminin from mouse Engelbreth-Holm-Swarm (EHS; Thermo Fisher Scientific) sarcoma (20 ng/µl), resulting in a final laminin density of 1–2 µg per cm^2^. The plates were then incubated overnight at 4 °C. GSCs were grown for 7 or 14 days into spheres; for some experiments, spheres were grown for 14 days in experimental pH conditions. The spheres were then gently spun down (400 g for 1.5 min) and resuspended in medium with different pH, with and without pharmacological inhibitors. Medium of ASIC2a over-expressing GSCs also contained 4-hydroxytamoxifen (4-HT). For 4-HT induction, the spheres had already been preincubated for 3 days with 4-HT at a concentration of 100 nM. Afterwards, a bystander blinded the experiment. The laminin was aspirated, the wells were washed once with PBS, and then the spheres were gently added into the wells, at a concentration that was empirically determined previously. The 24-well plate was then incubated at 37 °C, 5% CO_2_ for 5–6 h. Afterwards, the plates were carefully placed under the microscope and pictures taken with the IC Measure programme (The Imaging Source, Version 2.3.1). For experiments conducted under hypoxic conditions, the 24-well plates were incubated for 5–6 h in an Incucyte Live-Cell Analysis System (Sartorius AG, Göttingen, Germany), kindly provided by Dr. Jochen Maurer, at 37 °C, 5% CO_2_, < 3% O_2_. Pictures were taken from the Incucyte System with the built-in analysis system. Diameter of spheres and number of migrating cells were counted by hand and positions of cells marked, using ImageJ (NIH, Version 1.52a). Tumourspheres with a high background of freely migrating cells were disregarded, and only cells that could be clearly assigned to a tumoursphere were counted. Furthermore, only spheres with a diameter > 50 µm were quantified. After counting, the experiment was unblinded. The effect of ASICs on migration was tested using PcTx1 (Smartox Biotech, Saint-Egrevè, France), APETx2 (Alomone Labs, Jerusalem, Israel), or MitTx (Smartox Biotech) and the effect of KCNN4 and PI3K by TRAM-34 (Selleck Chemicals, Houston, USA) and wortmannin (Selleck Chemicals), respectively.

### Lentiviral transduction

A two-vector inducible system was used to generate ASIC2 overexpressing R54 cells. The cells were transduced first with a lentiviral vector pF GEV16 Super PGKHygro, which expresses a Gal4 DNA binding domain fused to a mutant oestrogen receptor and GEV16. Secondly, the cells were transduced with a lentiviral vector pF 5 × UAS W SV40 Puro ASIC2, which expresses ASIC2 in a Gal4-dependent manner. To generate lentiviral supernatants, 293 T cells were transfected with 3 mg pMD2.G, 5 mg pMDlg/pRRE, and 2.5 mg pRSV-Rev of the lentiviral packaging vectors [41] together with the constructs described above. The supernatants were harvested 48 h post-transfection, filtered (45 mm filter; Schleicher & Schuell, Keene, USA), and were added to R54 cells with 5 mg/ml polybrene, and R54 cells were spin-infected. Stable cell lines were selected in puromycin (1 mg/ml). The expression of ASIC2 was induced by exposure of the cells to 100 nM 4-hydroxytamoxifen for 24 h.

### Patch clamp

R54 GSCs overexpressing ASIC2 by lentiviral transduction were induced with 100 nM 4-HT 3 days before splitting. After splitting with 0.05% Trypsin/EDTA (Thermo Fisher Scientific), single cells were seeded on coverslips coated with poly-D-lysine and analysed by patch-clamp during the following 48 h. An Axopatch 200B amplifier and Digidata 1440A digitizer (Molecular Devices, San Jose, USA) controlled by Clampex (Version 10.6) were used for current recordings. The holding potential was − 70 mV. Micropipettes with a resistance of 4–6 MΩ were filled with an intracellular solution containing (in mM) 95 K-gluconate, 30 KCl, 1.2 NaH_2_PO_4_, 4.8 Na_2_HPO_4_, 5 glucose, 2.38 MgCl_2_, 1 EGTA, and 0.726 Ca^2+^-gluconate. The pH was adjusted to pH 7.2 using KOH and HCl. The cells were recorded in a perfused bath system at RT with an extracellular solution containing (in mM) 115 NaCl, 0.4 KH_2_PO_4_, 1.6 K_2_HPO_4_, 5 Glucose, 1 MgCl_2_, 25 Na^+^-gluconate, 3 Ca^2+^-gluconate, and 5 HEPES/MES. HEPES was used as a buffer for the standard bath solution of pH 7.3, and MES was used as a buffer for the stimulating bath solutions of pH 6 and 5. The pH-values were adjusted using NaOH and HCl. Each stimulation lasted 10 s. The cells were stimulated at least once with pH 6 before being activated with pH 5. Between stimulations, the cells were perfused with standard bath solution pH 7.3 for at least 60 s. To test the effect of PcTx1, 50 nM PcTx1 was added to the pH 6 and the pH 7.3 solution. It was pre-applied for 2 min. For analysis, the software ClampFit was used. Only fast activating (time to peak < 1.5 s) and transient currents with amplitudes > 10 pA were considered as ASIC-currents.

### Statistical analysis

Statistical analyses of qRT-PCR results were performed using two-sided *t* test in Prism 8 (Version 8.4.3), with significance threshold set to *p* ≤ 0.05.

For sphere migration assays (SMAs), the experimenter was blinded for the condition. We performed SMAs with at least three independent biological replicates with a variable number of technical replicates. To avoid pseudoreplication due to clustering of technical replicates from one biological replicate [28] and to account for the variable number of technical replicates, we statistically analysed SMAs using generalized linear mixed modelling (GLMM). GLMM was performed with RStudio “Prairie Trillium” (Build 461), using the packages glmmADMB [10, 44] and multcomp [19]. We neither saw zero-inflation nor overdispersion in our datasets and determined via Akaike information criterion and chi-square test that negative binomial distribution (migrated cells ~ treatment) provided the best fit as a generalized linear model for SMAs with 7-day old spheres. For SMAs with 14-day-old spheres, which were preconditioned in different pH, a nested model provided the best fit (migrated cells ~ preconditioning pH + preconditioning pH in interaction with assay pH). All models accounted for biological replicates as a random factor. Treatment conditions were then compared with a one-step Tukey’s all-pair comparison, using the glht function of the multcomp package, with significance threshold set to *padj* < 0.05.

Sphere diameters were analysed with one-way ANOVA in Prism 8 (Version 8.4.3), with significance set at *p* < 0.05. Normal distribution of the data for one-way ANOVA was confirmed via Anderson–Darling, D’Agostino-Pearson omnibus normality, Shapiro–Wilk normality and Kolmogorov–Smirnov normality tests in Prism 8 (Version 8.4.3), with significance threshold set to *p* < 0.05.

## Data Availability

Datasets generated and analysed during the course of this study are included in this published article. Additional information is available from the corresponding author upon reasonable request.
